# Prevalence and factors associated with the presence of non alcoholic fatty liver disease in an apparently healthy adult population in primary care units

**DOI:** 10.1186/1471-230X-7-41

**Published:** 2007-11-05

**Authors:** Llorenç Caballería, Ma Antonia Auladell, Pere Torán, Dolores Miranda, Jesús Aznar, Guillem Pera, Dolors Gil, Laura Muñoz, Jaume Planas, Santiago Canut, Jesús Bernad, Josep Aubà, Gregorio Pizarro, Miren Maite Aizpurua, Anna Altaba, Albert Tibau

**Affiliations:** 1 Primary Healthcare Centre Premià de Mar, Catalan Health Institute, IDIAP Jordi Gol, La Plaça 93,08330 Premià de Mar, Spain; 2 Primary Healthcare Research Support Unit Barcelonés Nord i Maresme. IDIAP Jordi Gol, Camí del Mig 36, 08303 Mataró, Spain; 3 Radiology Department, Primary Healthcare El Maresme, Catalan Health Institute, Camí del Mig 36, 08303 Mataró, Spain; 4 Radiology Department, Primary Healthcare Sant Adrià de Besós, Catalan Health Institute, C/Pl. Dr. Trueta s/n, 08930 Sant Adrià de Besós, Spain; 5 Primary Healthcare Centre Vilassar de Dalt, Catalan Health Institute, C/Plaça de la Vila 8, 08339 Vilassar de Dalt, Spain; 6 Primary Healthcare Centre Vilassar de Mar, Catalan Health Institute, C/Santa Maria 59-79, 08340 Vilassar de Mar, Spain; 7 Primary Healthcare Barcelonés Nord i Maresme, Catalan Health Institute, Sardana s/n, 08915 Badalona, Spain; 8 Primary Healthcare Centre Llefia, Catalan Health Institute, Carretera Antiga de Valencia s/n, 08913 Badalona, Spain; 9 Primary Healthcare Centre Gatassa, Catalan Health Institute, Camí del Mig 36 (4^a ^planta), 08303 Mataró, Spain; 10 Primary Healthcare Centre Gorg, Catalan Health Institute, Velez Rubio s/n, 08913 Badalona, Spain; 11 Primary Healthcare Centre Premià de Mar, Catalan Health Institute, La Plaça 93, 08330 Premià de Mar, Spain

## Abstract

**Background:**

Fatty liver disease is characterized by the accumulation of fat vacuoles inside of the hepatocytes. Non alcoholic fatty liver is associated with obesity, type 2 diabetes, dyslipemia, the intake of certain drugs and with the so-called metabolic syndrome. However, there is little information on the clinical relevance of this disorder as a healthcare problem in the general population, since the studies published generally include a limited number of patients and the diagnosis is established on the basis of clear biochemical alterations and liver biopsy.

**Methods/Design:**

The aim of the study is the prevalence of non-alcoholic fatty liver disease in a general adult population by hepatic ultrasonography.

A population-based, descriptive, transversal, multicentre study. Eighteen primary care centres of the north of Barcelona and the Maresme Areas of Healthcare Management attending an urban and semi-urban population of 360.000 inhabitants.

A randomized sample of 786 subjects of 15 years or older were selected from the population and assigned to the participating centres according to the Primary Care Information System (SIAP): This population is practically the same as the general population of the area.

The following determinations will be carried out in all the participants: hepatic ultrasonography to detect fatty liver, a questionnaire concerning liver diseases, alcohol intake, smoking and drug use, physical examination including abdominal perimeter and body mass index and biochemical analysis including liver function tests and parameters related to the metabolic syndrome and the HAIR score.

Ultrasonographic diagnosis of fatty liver will be made according to established criteria (American Gastroenterology Association) and diagnosis of metabolic syndrome according to the criteria of the European Group for the Study of Insulin Resistance.

**Discussion:**

This study will attempt to determine the prevalence of non alcoholic fatty liver disease, as well as, the factors most frequently associated with the presence of this disease to thereby achieve the most appropriate treatment and avoid the evolution of the disease.

## Background

Fatty liver disease is characterized by the accumulation of fat vacuoles in the cytoplasm of the hepatocytes, and is usually a mild lesion which is generally detected in chronic alcoholic patients [1].

Until the appearance of hepatic ultrasonography, fatty liver was a disorder diagnosed only by liver biopsy in individuals presenting hepatomegaly with or without biochemical abnormalities suggestive of liver disease [1,2].

At the beginning of the 1980s, a liver lesion was described which could not be differentiated from that associated with alcohol, and which covered a wide spectrum of patterns including simple fatty liver, steatohepatitis, with fatty vacuoles, necroinflammatory changes and a variable grade of fibrosis which may finally progress to liver cirrhosis in subjects without significant alcohol consumption [3,4]. Non alcoholic fatty liver is associated with factors such as obesity, type 2 diabetes, dyslipemia, the intake of certain drugs (amiodarone, oestrogens, and corticoids) and with the so-called metabolic syndrome (obesity, type 2 diabetes, dyslipemia, and arterial hypertension) [5-9].

Few studies have evaluated the prevalence of fatty liver in a general population. Along this line, the widest study undertaken was performed in a sample of almost 7.000 subjects in Northern Italy. In this study the most prevalent hepatic lesion was fatty liver (33% of the subjects with excessive alcohol intake) but ultrasonographic diagnosis was only performed in cases presenting clinical or analytical alterations suggestive of liver disease [10,11]. The same authors have recently published the results of another study with a new review of the prevalence and risk factors associated with fatty liver in the same series of patients and concluded that fatty liver is very prevalent (20%) in the general population and is associated with the different components of the metabolic syndrome [12]. Thus, non alcoholic fatty liver may be the hepatic component of the metabolic syndrome. Another study published in Spain attempted to make an epidemiologic approach to fatty liver by prospectively determining the prevalence, diagnosed by abdominal ultrasonography of 1703 working males and studying the relationship with alcohol intake and other risk factors such as obesity (body mass index > 30), hypercholesterolemia and hyperglycaemia and found that 13.8% fulfilled ultrasonographic criteria of fatty infiltration. Logistic regression analysis identified alcohol consumption > 40 g/day, age, obesity and elevated values of glycaemia, cholesterol and gammaglutamyltransferase as the main factors associated with the presence of fatty liver in this population [13].

More recently a population study has been published including 420 patients diagnosed with fatty liver not related to alcohol from 1980 to 2000 in different primary, secondary and tertiary healthcare centres in Minnesota. The authors precisely defined the morbidity and mortality associated with this disease. The patients had a mean age of 49 years, two out of three were obese and had hypertriglyceridemia, 36% were hypertensive, 26% had diabetes and 36% presented altered baseline glycaemia. The mean follow up of the patients was of 8 years. During this time period 13% of the patients died, representing a mortality greater than that expected in the general population (RR 1.34; p = 0.03). On multivariate analysis, the predictive factors of mortality were age, the presence of cirrhosis and surprisingly, altered baseline glycaemia. Liver disease constituted the third cause of death in these patients following neoplasms and ischaemic heart disease, in contrast with the general population in whom liver diseases are the thirteenth cause of death. Specifically speaking, during the follow up period, 5% of the patients were diagnosed with cirrhosis, 3% presented complications and 7 patients died in relation to their chronic liver disease (1.7%) [14].

The pathogenesis of non alcoholic fatty liver is only partially known, although it seems to be multifactorial. The most widespread pathogenic theory is that of the double impact in which the first is a resistance to peripheral insulin resulting in the accumulation of fat in the liver, and the second is chronic oxidative stress which leads to apoptosis and/or hepatocellular necrosis, inflammation and fibrosis [15,16].

As we have seen, the main risk factor for the development of non alcoholic fatty liver is, therefore, obesity. It is well known that the increase in the number of obese persons within the general population has progressed up to the point that the healthcare authorities consider it to be one of the main healthcare problems for the near future and making it one of the priorities for intervention and investigation. In addition, patients with non alcoholic fatty liver who are overweight and/or obese are more to likely to develop non alcoholic steatohepatitis, with a percentage of these patients evolving to more severe forms of liver disease. We therefore believe that it is important to determine the HAIR score established by Dixon et al [17] to know which patients may evolve to more severe forms of the disease. The HAIR score includes: arterial hypertension, insulin resistance defined as: type 2 diabetes with baseline glycaemia values greater than 110 mg/dl and less than 126 mg/dl and two of the following factors: arterial hypertension, triglycerides above 150 mg/dl and/or HDLc < 35 in males and < 39 in females, a waist/hip index > 0.90 in males and 0.85 in females and/or BMI > 30 Kg/m2, and ALT levels >40. Patients with a HAIR score ≥ 2 are considered to have a high probability of developing non alcoholic steatohepatitis.

The metabolic syndrome is constituted by the presence of a series of factors within the same subject such as obesity, arterial hypertension, dyslipemia or glucose intolerance and has been on the rise in recent years. Several criteria are used to define the metabolic syndrome, with the most commonly used being those of the World Health Organization (WHO) [18] and those of the Adult Treatment Panel III (ATP-III) of the National Cholesterol Education Program (NCEP) [19]. The criteria of the WHO require the presentation of some alteration in carbohydrate metabolism, whether diabetes, abnormal glucose tolerance or resistance to insulin. Two of the following criteria must also be taken into account: arterial hypertension > 140/90 mm Hg, obesity (BMI = 30 Kg/m^2^), hypertriglyceridemia ≥ 150 mg/dL or cHDL values < 35 in males and < 40 in females and microalbuminuria ≥ 20 μg/min. This definition places great importance on alterations in carbohydrate metabolism and insulin resistance as the elements necessary for the diagnosis of the metabolic syndrome. However, the definition by the WHO requires the presence of an alteration in glycaemia and does not place importance on the presence on abdominal obesity which is important to define insulin resistance. For this reason the European Group for the Study of Insulin Resistance (EGIR) has proposed several changes in the definition of the WHO, such as the presence of obesity (waist perimeter ≥ 94 cm), fasting plasma insulin determination and altered fasting glycaemia [20]. The criteria of ATP-III of the NCEP are based on the presence of abdominal obesity (≥ 88 cm in women and ≥ 102 cm in males), hypertriglyceridemia (≥ 150 mg/dL), low cHDL plasma values (<35 mh/dL), arterial hypertension (≥ 130/85 mm Hg) and altered fasting glycaemia (≥ 110 mg/dL). The criteria of the ATP-III are more clinical and easier to apply than those of the WHO, but the results of several studies have shown that the ATP-III criteria overestimate the presence of the metabolic syndrome. On the other hand, the criteria of the ATP-III do not correlate with those using insulin resistance as the fundamental element of diagnosis. Given the importance of the metabolic syndrome in the diagnosis of non alcoholic fatty liver and the discrepancies between the two models, we believe it may be useful to analyze both models in all the subjects to determine wihch model best defines the metabolic syndrome.

### Objectives

#### Main objectives

1. Determine the prevalence of non alcoholic fatty liver in apparently healthy adults who do not request medical care and who have undergone a hepatic ultrasongraphy.

2. Describe the associated characteristics of these subjects based on: sociodemographic variables, history of liver disease and associated risk factors.

3. Evaluate the influence of each of the components making up the metabolic syndrome and the risk of having non alcoholic fatty liver.

4. Identify the subjects who may progress to chronicity and the factors favouring progresión.

#### Secondary objectives

1. Identify a cohort of patients with fatty liver with no evidence of previous liver disease to perform a prospective follow up.

## Methods/Design

We will carry out a descriptive, transversal, population-based, multicentre study in apparently healthy adults (older than or equal to 15 years of age) or subjects not requesting medical care who have undergone a hepatic ultrasonography. The study has been approved by the Ethical Committee of Clinical Investigation, Jordi Gol i Gurina Foundation.

### Study subjects

This will be a multicentre study including the participation of Primary Care teams covering a population of 360,000 inhabitants of an urban, and semi-rural zone of the North of Barcelona and the Maresme, Spain.

#### Inclusion criteria

Adult population of both sexes from these Primary Care teams between the age of 15 and 80 who wish to voluntarily participate in the study and who have signed a written informed consent form to participate.

#### Exclusion criteria

Alcohol intake > 30 g/day in males and > 20 g/day in females. Presence of chronic liver disease. Presence of the surface antigen of the hepatitis B virus or the presence of antibodies versus hepatitis C. Subjects with conditions or diseases hindering data collection and follow up of the study such as, incapacitating diseases, cognitive deterioration, institutionalized patients or subjects with no fixed address in any of the basic areas of the study. Subjects who do not provide written informed consent to participate in the study.

### Sample size

With an alpha risk of 0.05 for a precision of ± 0.03 percentage units in a bilateral contrast for an estimated proportion of 0.20 a randomized sample proportional to 683 subjects is required. Considering that 15% of the population will present alcohol intake or some previous liver disease, the number of patients to study in this percentage is increased making a total of 786 individuals necessary to fulfil the final sample size.

### Selection method

A randomized sample of the population assigned and obtained through the SIAP (Primary Care Information System), will be stratified in proportion to the population assigned to each of the Primary Care teams participating in the study. It should be pointed out that the population assigned is practically equivalent to the updated municipal pattern.

### Phase I

The resulting variable is the ultrasonographic diagnosis of fatty liver. The diagnosis of fatty liver will be established according to the standard criteria accepted by the American Gastroenterology Association [21]: An increase in hepatic echogenicity taking renal echogenicity as a reference, the presence of enhancement and lack of differentiation in periportal intensity and the vesicular wall due to great hyperechogenicity of the parenchyma. The degree of involvement will be standardized with a semiquantitative scale of the degree of hepatic enhancement.

The following covariables will be evaluated in the study subjects:

1. Sociodemographic variables: age, sex, occupation, education and place of residence.

2. Clinical history including:

2.1 Personal history of: liver disease, biliary lithiasis, surgical interventions.

2.2 Presence of comorbidities: obesity and being overweight, type 2 diabetes, dyslipemia and arterial hypertension.

2.3 Alcohol intake: determine the type and quantity of beverages consumed.

2.4 Smoking habit.

2.5 Habitual drug use: history of drug use during the previous six months.

3. Physical examination:

3.1 Antropometric data: weight, height, abdominal obesity and body mass index.

3.2 Determination of arterial pressure.

4. Analytical determinations:

4.1 Blood analysis including: a complete hemogram, glycaemia, glycosylated haemoglobyn, urea, creatinine, uric acid, a lipid study (cholesterol, triglycerides, HDL, LDL) and liver function tests, including hepatitis markers (surface antigen of the hepatitis B virus and antibodies versus the hepatitis C virus).

4.2 Baseline insulin levels will be determined by the immunochemoluminescence method.

4.3 Determination of insulin resistance will be estalished with the HOMA method (homeostasis model assessment). ([glycaemia(mmol/L)/insulin (mU/L)]/22.5).

4.4 A sample of the first urine in the morning will be taken to determine microalbuminuria.

5. Determination of the HAIR score [17].

6. Diagnosis of the metabolic syndrome: This will be made according to the criteria of the WHO [18] modified by the European Group for the Study of Insulin Resistance [20], and the National Cholesterol Education Program (NCEP) [19].

### Phase II

Patients with diagnosed liver disease will receive the normal control and follow up by their reference physician. Patients with fatty liver with no evidence of liver disease will be advised to modify their habits and risk factors and will initiate annual control and ultgrasonographic follow up. The resulting variable in this phase will be the persistence, reversal or progression of liver involvement. This subgroup of subjects will provide a cohort of patients to obtain data on the natural history of the disease in future studies.

### Plan of analysis

Data will be introduced into an ACCESS type database and then analysed. The prevalence of fatty liver will be estimated with a confidence interval of 95%.

A descriptive univariate and bivariate statistical analysis will be performed, analysing the qualitative variables with chi-squared test and the quantitative variables with the Students-t- test. Statistical significance will be established at 5% for all the bivariate analyses and contrasts will be considered at a bilateral level. Multivariate analysis will also be undertaken with logistic regression models (dependent variable presence/absence of fatty liver) allowing adjustment of the effect of the different independent variables on the presence of non alcoholic fatty liver.

## Discussion

Non alcoholic fatty liver is possibly the most common cause of an elevation in transaminases in adults [22]. Taking into account the progressive increase in the prevalence of obesity, type 2 diabetes, and dyslipemia in the population, studies performed in the USA consider that the prevalence of non alcoholic fatty liver would be much higher than the prevalence of hepatitis C virus infection estimated to be 1.8% [7]. With the increase in the prevalence of these associated diseases, non alcoholic fatty liver may become one of the most frequent causes of chronic liver disease in Western countries [23].

As a consequence of periodic health examinations in the general population and the greater accessibility to diagnostic imaging tests in primary care, minimum hepatic biochemical alterations are increasingly found which are only correlated with an ultrasonographic diagnosis of fatty liver. In Spain the diagnosis of non alcoholic fatty liver is increasingly more frequent due to the interest given and the increase in obesity, manifesting during adolescence. Thus, if it used to be considered a disease mainly affecting middle-aged, obese, and in many cases diabetic, women, it has been shown to affect both sexes equally after adolescence [24].

Another of the limitations of the study is related to the use of hepatic ultrasonography for the diagnosis of fatty liver. The gold standard for diagnosis of this disease is liver biopsy. Nonetheless, studies comparing the diagnostic utility of ultrasonography compared with liver biopsy have shown a sensitivity of greater than 90% and a specificity of greater than 80% for ultrasonography in detecting the presence of fatty liver. The main limitation seems to be the difficulty in detecting the presence of fatty liver when the infiltration is of less than 30% of the hepatic content. Another limitation of ultrasonography is the lack of information regarding the histologic changes associated with disease progression. To achieve this liver biopsy would be required which, in our case would be referred to the reference hospital on both clinical and biochemical suspicion of progression. Therefore, ultrasonography of the liver is currently the test of reference for the detection of fatty liver at a population level.

Although increasingly more knowledge on this disease has been obtained, several important aspects remain to be elucidated, such as the establishment of the real prevalence of this disease, the identification of the cases which may progress to chronicity, the factors favouring progression, the search for non invasive methods to detect the presence of fibrosis and clinical trials with the different treatments which have been proposed.

To date no study of these characteristics has been undertaken in Primary Care and in a population base in Spain. Thus, a study such as this may have important social, healthcare and economic impact since it will allow the estimation of the real presence of a silent healthcare problem with potentially severe consequences which may, in part, be avoided by early intervention. These defining characteristics (high prevalence and possibility of early intervention) making non alcoholic fatty liver a healthcare problem which should, typically, be approached from Primary Care but which seems to be under-evaluated and underdiagnosed. This is the framework within which this project is integrated: the estimation of the prevalence and quantification of the underdiagnosis in the general population.

## Competing interests

The author(s) declare that they have no competing interests.

## Authors' contributions

LCR, JALL, PTM, GPR, AAB, M^a^AALL, LMO and GPB participated in the design of the study; LCR, GPR, AAB, M^a^AALL, ATC, MAP, SCC and JBS contributed to the coordination study; DMB, JAC, JPP, DGM performed the ecographies; GPB participated in the statistical calculations. All the authors have read, revised and approved the final manuscript.

## Pre-publication history

The pre-publication history for this paper can be accessed here:


